# Solid-Liquid Separation Properties of Thermoregulated Dicationic Ionic Liquid as Extractant of Dyes from Aqueous Solution

**DOI:** 10.1155/2018/3980364

**Published:** 2018-06-03

**Authors:** Rui Lv, Shuya Cui, Yangmei Zou, Li Zheng

**Affiliations:** Department of Chemistry and Chemical Engineering, Mianyang Teacher's College, Mianyang, Sichuan 621000, China

## Abstract

Two thermoregulated dicationic ionic liquids were synthesized and applied for effective extraction of the common dye malachite green oxalate (MG). The extraction parameters such as amount of ionic liquids, pH of water phase, extraction time, cooling time, and centrifugal time on the extraction efficiency were investigated systematically. It revealed that the dye has been successfully extracted into the ionic liquids, with high extraction efficiency higher than 98%, and recovery of 98.2%–100.8%, respectively. Furthermore, these ionic liquids can be recycled easily after elution. The reusable yields were 87.1% and 88.7%. The extraction of the dye into the thermoregulated ionic liquid provides a method of minimizing pollution of waste water potentially.

## 1. Introduction

Ionic liquid is a widely used extraction solvent, because of its high heat stability, vapor pressure, nonvolatility, and friendly environment perception [1]. In recent years, there are many researches about ionic liquid as an extractant for dye from various samples such as Congo red, Sudan dye, methyl orange, and so on [2–4], but the application of ionic liquids is restrained due to its high cost and hard to recycle [5, 6].

Thermoregulated ionic liquids are a kind of ionic liquids, with appearing liquid phase at high temperatures and solid phase at low temperatures [7]. They can be used to achieve separation and enrichment of the target compounds by changing the temperature. Further, based on the advantages, it can be easily recycled. In recent years, the reports about using temperature-sensitive ionic liquids as extractant have been mostly concentrated in the area of metal ions [8, 9], but rarely involved in dyes.

Malachite green (MG), which is one of the triphenylmethane dyes, is widely used in aquaculture to prevent fungal infections and kill parasites due to its low price. However, it was found that malachite green had potential carcinogenic, teratogenic, and mutagenic effects [10]. So, it was banned to use in aquaculture in several countries in the world [11]. Therefore, separation and determination of malachite green in water is very important to human health.

As the residues of malachite green in water are low, the efficient enrichment method is needed. Common methods of separation and enrichment are physical adsorption [12, 13], liquid-liquid extraction [14, 15], and solid-phase extraction (SPE) [16, 17]. These methods have lots of disadvantages to limit their application because they are expensive or use organic solvent which may lead to environmental pollution. Therefore, looking for a simple, efficient green method becomes necessary.

In this study, two thermoregulated ionic liquids with hydrophobic properties were synthesized and employed to separate and concentrate malachite green in water. Some parameters which would influence the separation process were investigated, and the optimum condition was obtained. Further, the feasibility of the method was validated by analyzing the real water sample and simulation water sample. The efficient, simple, and environmental friendly method was confirmed to be useful to separate and enrich the malachite green in water.

## 2. Materials and Methods

### 2.1. Chemicals

Standard malachite green was supplied by the National Research Center for Reference Material (Beijing, China). N-methylimidazole (>99%) and KPF_6_ (>99%) were purchased from Shanghai Aladdin Reagent Co., Ltd. (Shanghai, China). Thionyl chloride, diethylene glycol, triethylene glycol, Na_2_HPO_4_, and NaH_2_PO_4_ were all of analytical grade and brought from Chengdu Kelong Reagent Co., Ltd. (Sichuan, China).

### 2.2. Apparatus

The melting point of ionic liquid was tested by XT-4 melting point meter (Beijing Tech Instrument Co., Ltd.) at room temperature with the thermometer uncorrected. ^1^H NMR and ^13^C NMR spectra were recorded on 600 MHz NMR spectrometer (Varian, USA) with the solvent of acetone-d. FTIR spectra were registered on IRAffinity-1S FTIR spectrometer (Shimadzu, Japan). UV-visible spectra were obtained from a T6 UV-visible spectrophotometer (Beijing Persee, China) at the maximum absorption wavelength of 616.9 nm. TG16-W high-speed centrifugation (Hunan Xiangyi Laboratory Instrument Development Co., Ltd., China) was employed to accelerate the phase separation process. The temperature was controlled by HH-6 thermostatic water bath (Changzhou Jintan Liangyou Instrument Co., Ltd., China). The pH of the extraction system was measured with PHSJ-3F pH meter (Shanghai Inesa Analytical Instrument Co., Ltd., China).

### 2.3. Water Sample Collection

Water samples were collected from fish ponds in Mianyang, China. All the water samples were filtered through 0.45 *μ*m microporous membrane filter before extraction.

### 2.4. Preparation of Ionic Liquids

With diethylene glycol-bridged functionalized imidazolium dicationic ionic liquids as an example, synthesis route of polyethylene glycol-bridged functionalized imidazolium dicationic ionic liquids was shown in four steps as follows: Firstly, thionyl chloride was added to diethylene glycol in anhydrous environment and then heated to 250°C until no starting material was observed using TLC. And then, it was cooled and extracted with CHCl_2_. The extract was washed with water, saturated NaHCO_3_ solution, and saturated NaCl solution. The product is dried under reduced pressure. Secondly, N-methylimidazole was added to the above dichlorodiglycol in methanol at room temperature. The reaction continued for 24 h. The solvent was removed under reduced pressure to yield a viscous gum and soluble in water and in methanol. The obtained dichloride salt was used directly, without further purification. Then, the obtained dichloride salt was dissolved in methanol, and then, KPF_6_ was added with rapid stirring. After four hours, the solvent was removed under reduced pressure. The residue was washed by water and filtrated to collect the solid. The solid was recrystallized in acetone and the colorless crystal generated, named IL-1 and 2IL-2. For IL-1, the yield was 90%. m.p.: 91–93°C. IR (*ν*max, KBr, cm^−1^): 3174, 2916, 1627, 1577, 1460, 1324, 1161, 850, 557. ^1^H NMR (600 MHz, CD_3_COCD_3_, *δ*, ppm): 3.97 (s, 6H, N–CH_3_), 4.05 (4H, s, N–CH_2_), 4.32 (4H, s, O–CH_2_), 7.68 (4H, C (4,5)–H), 9.03 (2H, C(2)–H); ^13^C NMR (150 MHz, CD_3_COCD_3_, *δ*, ppm): 136.7 (C-2), 124.5, 124.0 (C-4,5), 69.5(O–CH_2_), 50.3 (N–CH_2_), and 36.6 (N–CH_3_). The structure of IL-2 was consistent with the data reported by the previous literature [18].

**Figure 1:**
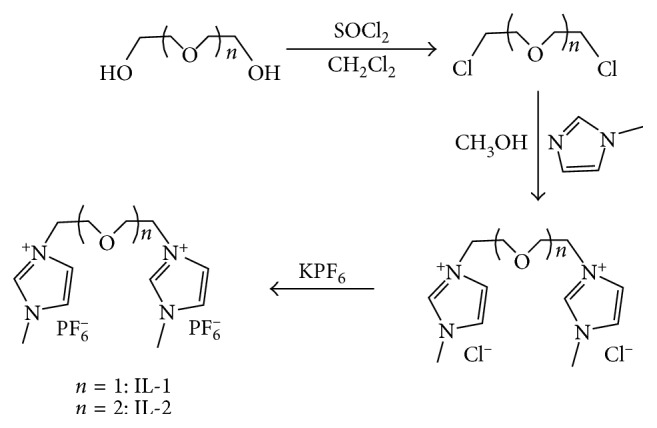
Synthesis route of two thermoregulated dicationic ionic liquids.

The synthesis route and structures of these two thermoregulated dicationic ionic liquids are shown in Figure 1.

### 2.5. Preparation of Standard Solution

A stock solution of malachite green (0.5 wt.%) was prepared by dissolving in distilled water. Other concentrations of the sample solution were prepared by diluting the stock solution with distilled water.

### 2.6. Experimental Methods

The extraction experiment was carried out in the sample solution with two ionic liquids (IL-1 and IL-2) as extractants, respectively. Firstly, mix the sample solution and ionic liquid and heat the mixture solution to make the ionic liquid melt and distribute in the sample solution for extraction (90°C for IL-1 solution and 60°C for IL-2). After that, the mixture was cooled to room temperature while the IL was separated. Finally, the mixture was centrifuged at 3000 r/min to get the supernatants, and the concentration of dye was measured using UV spectrophotometer (616.9 nm). The extraction ratio can be calculated by the following equation:

(1) $$\begin{array}{c} R\left(\%\right)=\frac{\left(C_{0}-C_{1}\right)V_{\mathrm{aq}}}{C_{0}V_{\mathrm{aq}}}\times 100\%, \end{array}$$

where *C*_0_ represents the concentration of malachite green in the sample solution, *C*_1_ refers to the concentration of malachite green in supernatants after extraction, and *V*_aq_ is the volume of the extraction phase. *C*_1_ was obtained by standard curve calculation.

## 3. Results and Discussion

### 3.1. Effect of the Amount of Ionic Liquids

**Figure 2:**
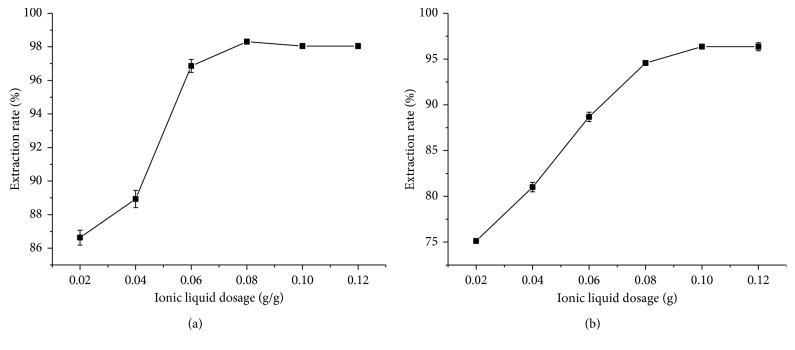
Effect of the amount of ionic liquids on extraction rate. (a) IL-1; (b) IL-2. The error bars represent the standard deviation of measurements in three parallel experiments (*n*=3).

The amount of extractive agent was an important parameter for sample extraction efficiency. The effect of the amount of ionic liquid on the extraction rate was examined and the result was shown in Figure 2. It was found that the extraction rate reached the maximum of 98.39% when the weight of IL-1 increased to 0.0800 g, while the maximum extraction rate was 96.35% when the IL-2 was 0.1000 g. Therefore, 0.0800 g IL-1 and 0.1000 g IL-2 were chosen, respectively, for the following experiments.

### 3.2. Effect of pH

**Figure 3:**
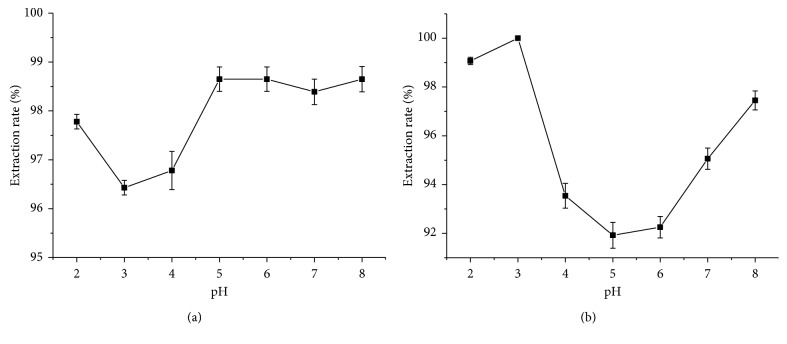
Effect of the pH on extraction rate. (a) IL-1; (b) IL-2. The error bars represent the standard deviation of measurements in three parallel experiments (*n*=3).

The structure of malachite green varied with pH in solution, which affected the extraction rate of ionic liquids. Phosphate buffer was applied to control pH values. The effect of pH on the extraction rate was determined (Figure 3). Results showed that the extraction rate was varying slightly with pH when IL-1 was employed as the extractant; by comparison, when IL-2 was employed as the extractant, the extraction rate first increased and then decreased with the increase in pH. But both of them can obtain the optimum extraction efficiency in the acidic solution. It may be because the p*K*a of MG is 6.9; when the pH was lower than it, the MG presents in its molecular state, which may help in extraction. But owing to the different molecular space conformation in the two ILs, they may have different effects depending on the pH value. Therefore, pH 5 was chosen as the optimal pH for IL-1, and pH 3 was chosen as the optimal pH for IL-2.

### 3.3. Effect of Extraction Time

Ionic liquids show the liquid state when the extraction temperature was set above their melting point. Under this temperature, the target compounds in water can be extracted. Therefore, in this study, the extraction temperature was set at 90°C for IL-1, while it was set at 60°C for IL-2, respectively, to investigate the effects of extraction time (2–12 min with 2 min interval) on the extraction rate. From the results, it was found that when IL-1 was used as an extraction agent, the extraction rate reached the maximum when the extraction time was 4 min. At the same time, when IL-2 was used as an extraction agent, the extraction rate can reach 100% when the time was 1 min.

### 3.4. Effect of Cooling Time

For thermoregulated ionic liquid, when the temperature is below its melting point, it will be a hydrophobic solid precipitation, and cooling time determines the amount of precipitation. So, the effect of cooling time (1–11 min with 2 min interval) on extraction was studied. The experiment results showed that when the cooling time was 3 min for IL-1 and 7 min for IL-2, it reached the maximum extraction rate.

### 3.5. Effect of Centrifugal Time

The centrifugal process is to separate the solid extraction reagent and sample solution. The effect of centrifugal time (5–15 min with 5 min interval) on the extraction rate was investigated. It was found that when centrifuged at 3000 r/min for 5 min, the extraction rate reached the maximum.

**Figure 4:**
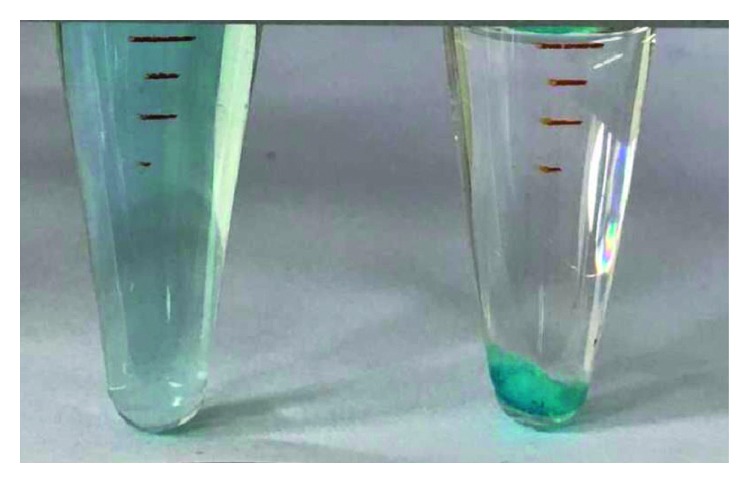
The ionic liquid at extraction stage and separation stage.

In summary, the optimum condition for IL-1 was as follows: 0.0800 g IL, buffer pH 5, extraction time 4 min, cooling time 3 min, and centrifugation at 3000 r/min for 5 min, and the optimum condition for IL-2 was 0.1000*g*, pH 3, extraction time 1 min, cooling time 7 min, and centrifugation time 5 min at 3000 r/min. Under the optimum conditions, the extraction rate of malachite green was 98.90% and 100% by using IL-1 and IL-2, respectively.  Figure 4 shows the ionic liquid at the extraction stage and separation stage under the optimum conditions.

### 3.6. Influence of Interference Pigments

Effects of presence of methylene blue on extraction of malachite green by two ionic liquids were observed under the optimized condition. Results showed that the extraction rates of malachite green were more than 95% in presence of interference pigments, which indicated that these ionic liquids have good selectivity for extracting malachite green.

### 3.7. Recycling of Ionic Liquid

These ionic liquids can be recycled easily after elution. On addition of 1 mol/L NaOH to the ionic liquids after extraction, malachite green was removed from the mixture. The recycle yields were 87.1% and 88.7% for IL-1 and IL-2, respectively.

### 3.8. Linearity, Precision, and Accuracy

Under the optimized conditions, the absorbance of malachite green increased linearly in the range of 0–2.5 mg·L^−1^. The linear regression equation was *A* = 0.1563C–0.0023, and the correlation coefficient (*R*^2^) was 0.9999. Moreover, the limit of detection was calculated to be 0.044 mg·L^−1^.

Under the optimal experimental conditions, the relative standard deviation (RSD) of the method was determined by using 5 mg/L malachite green standard solution for 6 times, with 0.23% for IL-1 and 0.37% for IL-2, respectively. The results showed this method has better accuracy.

Experimental results with the recoveries obtained by the standard addition method are shown in Tables 1 and 2. The recovery results were in the range from 98.20% to 100.80%. From these results, it was found that the method has good accuracy and it can be used for extraction of malachite green accurately.

## 4. Conclusions

In this study, two thermoregulated ionic liquids were firstly employed as extractants to separate and concentrate malachite green in water. The optimum extraction conditions were achieved with the extraction rate of approximately 100%. Two ionic liquids can be recycled after the elution process, with the recovery higher than 87%. The method provided in this study is not only simple and efficient, selective for extraction of malachite green in the water, but also can avoid the environmental pollution by the extraction agent.

## Figures and Tables

**Table 1:** The recovery test results of extraction by IL-1.

Sample	Origin (mg/L)	Added (mg/L)	Found (mg/L)	Recovery (%)	RSD
Water sample	0	5	4.91	98.20	0.00
		10	9.88	98.80	0.09
		20	19.83	99.15	0.00

Simulation water sample	9.93	5	14.92	99.74	0.11
		10	19.91	99.74	0.00
		20	29.88	99.74	0.11

**Table 2:** The recovery test results of extraction by IL-2.

Sample	Origin (mg/L)	Added (mg/L)	Found (mg/L)	Recovery (%)	RSD
Water sample	0	5	4.91	98.20	0.00
		10	9.88	98.80	0.09
		20	19.92	99.60	0.00

Simulation water sample	9.60	5	14.64	100.80	0.11
		10	19.59	99.90	0.00
		20	29.50	99.50	0.11

## Data Availability

The data used to support the findings of this study are available from the corresponding author upon request.
